# Diabetic foot infections: Application of a nisin-biogel to complement the activity of conventional antibiotics and antiseptics against *Staphylococcus aureus* biofilms

**DOI:** 10.1371/journal.pone.0220000

**Published:** 2019-07-24

**Authors:** Raquel Santos, Diana Ruza, Eva Cunha, Luís Tavares, Manuela Oliveira

**Affiliations:** CIISA-Centro de Investigação Interdisciplinar em Sanidade Animal, Faculdade de Medicina Veterinária, Universidade de Lisboa, Lisboa, Portugal; High Point University, UNITED STATES

## Abstract

**Background:**

Diabetic foot infections (DFIs) are a frequent complication of Diabetes *mellitus* and a major cause of nontraumatic limb amputations. The Gram-positive bacterium *Staphylococcus aureus*, known for its resilient biofilms and antibiotic resistant profile, is the most frequent DFI pathogen. It is urgent to develop innovative treatments for these infections, being the antimicrobial peptide (AMP) nisin a potential candidate. We have previously proposed the use of a guar gum biogel as a delivery system for nisin. Here, we evaluated the potential of the nisin-biogel to enhance the efficacy of conventional antibiotics and antiseptics against DFIs *S*. *aureus* clinical isolates.

**Methods:**

A collection of 23 *S*. *aureus* strains isolated from DFI patients, including multidrug- and methicillin-resistant strains, was used. The antimicrobial activity of the nisin-biogel was tested alone and in different combinations with the antiseptic chlorhexidine and the antibiotics clindamycin, gentamicin and vancomycin. Isolates’ *in vitro* susceptibility to the different protocols was assessed using broth microdilution methods in order to determine their ability to inhibit and/or eradicate established *S*. *aureus* biofilms. Antimicrobials were added to the 96-well plates every 8 h to simulate a typical DFI treatment protocol. Statistical analysis was conducted using RCBD ANOVA in SPSS.

**Results:**

The nisin-biogel showed a high antibacterial activity against biofilms formed by DFI *S*. *aureus*. The combined protocol using nisin-biogel and chlorhexidine presented the highest efficacy in biofilm formation inhibition, significantly higher (p<0.05) than the ones presented by the antibiotics-based protocols tested. Regarding biofilm eradication, there were no significant differences (p>0.05) between the activity of the combination nisin-biogel plus chlorhexidine and the conventional antibiotic-based protocols.

**Conclusions:**

Results provide a valuable contribution for the development of complementary strategies to conventional antibiotics protocols. A combined protocol including chlorhexidine and nisin-biogel could be potentially applied in medical centres, contributing for the reduction of antibiotic administration, selection pressure on DFI pathogens and resistance strains dissemination.

## Introduction

Diabetes *mellitus* (DM) is a chronic disease that affects more than 422 million people worldwide. Moreover, in the recent decades, the prevalence of DM has increased from 4.7% in 1980 to 8.5% in 2014 [1]. As a consequence, DM-associated foot ulcers (DFUs) prevalence has also increased [2]. These ulcers result from consequence of a complex interaction of several pathophysiological factors, mainly neuropathy, vasculopathy and immunopathy [3], being observed that approximately 15 to 25% of patients with DM develop DFUs in their lifetime [4].

Around half of DFUs become clinically infected, usually by opportunistic pathogens [5]. Diabetic foot infections (DFIs) are a frequent and complex problem that causes severe morbidity, including distress, and reduced physical and psychological quality of life. DFI treatment requires wound care, antimicrobial therapy, and often surgical procedures [2]. As a result, DFIs are the most common diabetic complication requiring hospitalization and the world’s leading cause of nontraumatic lower extremity amputation [6].

DFIs are caused by a polymicrobial community of pathogens, mainly formed by Gram-positive bacteria, with *Staphylococcus aureus* being the most prevalent species [4–5,7]. This commensal bacterium is known to asymptomatically colonize the human skin and mucosal surfaces, being permanently present in 20 to 30% of the population, while other 30% are transient carriers [8].

*S*. *aureus* is recognized for its ability to develop resistance to different antibiotic classes and infections caused by antibiotic resistant *S*. *aureus* strains are globally reaching epidemic proportions [9]. In fact, a key problem in DFI treatment is the increasing incidence of antibiotic resistant pathogens, particularly Methicillin-Resistant *S*. *aureus* (MRSA) [10–11]. Among hospitalized patients, the prevalence of MRSA in DFIs can range from 15 to 30% [4].

Another important *S*. *aureus* virulence factor responsible for antibiotic therapeutic failure in DFIs is the formation of biofilms [12]. These slime-enclosed aggregates of sessile bacteria are embedded within a self-produced matrix of extracellular polymeric substances and irreversibly attached to surfaces [13]. Due to ineffective diffusion or sequestering of antimicrobial agents within the biofilm, these bacterial communities demonstrate great resistance to most antibacterial agents as well as to host defenses [14].

Currently, the treatment of infected DFUs consists of surgical debridement followed by wound cleansing with an antiseptic solution and antibiotics administration [6]. A wide variety of antiseptics is available, being chlorhexidine one of the most frequently used in DFIs [15]. It is widely used worldwide for skin antisepsis and daily skin cleansing with chlorhexidine has been used to control *S*. *aureus* infections, including MRSA outbreaks [16]. Additionally, chlorhexidine has also shown some ability to inhibit microorganism’s adherence to surfaces, thereby preventing the growth and development of biofilms [17–18].

Antibiotics administration for DFI treatment can be performed oral or intravenously, depending on the severity of infection. According to the guidelines for the medical management of DFI from Lipsky et al., [2,6], Chidiac et al., [19], Bader [20], and Duarte and Gonçalves [21], the antibiotics of choice for mild, moderate and severe DFI are, respectively, clindamycin (450 mg, 8/8h, oral), gentamicin (5 mg/kg, 24/24h, intravenous) and vancomycin (30 mg/kg, 12/12h, intravenous).

Clindamycin has been considered a first line choice for the treatment of various skin and soft tissue infections, like DFIs. It can also be used for the treatment of moderate and severe DFI, but in such cases it should be combined with other antibiotics from different classes [2,19–20], Gentamicin is commonly used for the prophylaxis and treatment of moderate and severe DFI [19,21], while vancomycin use is reserved for cases of severe infection, being considered a last resource antibiotic against MRSA infections [22].

As the DFI treatments available are often ineffective [23], new therapeutic strategies for DFI treatment are urgent and the application of topical antimicrobial peptides (AMPs) may be a useful complement or alternative to conventional treatments. These molecules are produced by living organisms as part of their immune response against pathogens [24], can act as modulators of the immune system [25], and are able to prevent biofilm formation and act on pre-formed biofilms [26–27], supporting their potential as DFI therapeutic agents.

Nisin is an AMP produced by *Lactococcus lactis*, whose spectrum of activity includes a wide range of Gram-positive bacteria, including *S*. *aureus* [27–28]. In 1969, this bacteriocin was considered safe for use as a food preservative by the Food and Agriculture Organization and World Health Organization, being also approved by the US Food and Drug Administration in 1988. Nowadays, it is used in over 48 countries [29].

Considering that AMPs can be degraded or inactivated before reaching their target at therapeutic concentrations [30], it is mandatory to establish effective AMP delivery systems, with the natural polysaccharide guar gum being one of the most promising [27]. A previous work conducted by our team demonstrated that a biogel formed by nisin incorporated in guar gum not only presented a high level of antimicrobial activity against planktonic *S*. *aureus* DFI isolates, but most importantly, it was able to inhibit and eradicate biofilm-based bacteria, including those formed by MRSA and MDR clinical strains [27].

Although AMPs represent a potential novel strategy for DFI treatment, conventional antibiotics remain the standard therapeutic protocols and cannot be fully replaced at the present. Considering that AMPs can be used in combination with antibiotics [31], this work aimed at evaluating the potential of the previously developed nisin-biogel [27] in enhancing the efficacy of DFI treatment based on conventional antibiotics and antiseptics, using *S*. *aureus* clinical isolates as bacterial models, and an innovative protocol to simulate *in vitro* the application of currently accepted DFI therapeutic protocols.

## Materials and methods

### Bacterial strains

Isolates were obtained in a previous epidemiological survey regarding DFU infections, conducted at 4 clinical centers in Lisbon from January to June 2010 [5]. A total of 53 staphylococci were collected from 49 DFU patients, from which 23 representative biofilm-producing *S*. *aureus* isolates were selected, based on Pulse Field Gel Electrophoresis (PFGE) and Multilocus Sequence Type (MLST) profiling [10]. In addition, a biofilm-producing reference strain, *S*. *aureus* ATCC 29213, was also included in this study as a control strain.

The antimicrobial resistance profile of these strains was previously characterized through determination of the Minimal Inhibitory Concentration (MIC) for ten antibiotics and by multiplex PCR for detection of the following genes: *mecA*, *mecC*, *erma*, *ermB*, *ermC*, *blaZ*, *msrA*, *aac-aph*, *tetK*, *tetL*, *tetM*, *tetO* and *norA*. It was observed that 35% (n = 8) of the isolates were MRSA and 30% (n = 7) were considered to be multidrug resistant [10]. All of these strains (n = 23) were classified as biofilm-producers [32].

Isolates were stored at -80°C in buffered peptone water supplemented with 20% (v/v) of glycerol.

### Chlorhexidine Minimum Inhibitory (MIC) and Bactericidal (MBC) Concentrations

Strains were grown in a non-selective Brain Heart Infusion (BHI) agar medium (VWR, Belgium) at 37°C for 24 h. Bacterial suspensions of approximately 10^8^ c.f.u./mL were prepared directly from plate cultures using a 0.5 McFarland standard (bioMèrieux, France) in sterile normal saline (Scharlau, Spain). Afterwards, bacterial suspensions were diluted in fresh BHI broth to a concentration of 10^7^ c.f.u./mL.

A stock solution of chlorhexidine at 4% (w/v) (AGA, Portugal) was filtered using a 0.22 μm cellulose acetate membrane filter (VWR, Belgium) and diluted in sterile water to obtain a set of solutions with concentrations ranging from 0.15 to 70 μg/mL. Solutions were stored protected from the light at 22°C until use.

The set of chlorhexidine solutions were distributed in 96-well flat-bottomed polystyrene microtitre plates (Nunc; Thermo Fisher Scientific, Denmark). All the wells, except for the negative control (with broth medium only), were inoculated with 150 μL of the 10^7^ c.f.u./mL bacterial suspensions. Microplates were incubated statically for 24 h at 37°C, and MIC was determined as the lowest concentration of chlorhexidine that visually inhibited bacterial growth [33].

MBC value was determined by inoculating on BHI agar plates 3 μL of the suspensions from the wells where no bacterial growth was observed. Plates were incubated at 37°C for 24 h and MBC was determined as the lowest chlorhexidine concentration from which no bacterial colonies were observed [34].

Experiments were conducted in triplicate, and independent replicates were performed at least three times in different days.

### Antimicrobials solutions

A stock solution of nisin (1000 μg/mL) was obtained by dissolving 1 g of nisin powder (2.5% purity Sigma-Aldrich, USA) in 25 mL of HCl (0.02 M) (Merck, Germany), filtered using a 0.22 μm cellulose acetate membrane filter and stored at 4°C. The stock solution was then diluted with sterile water to a concentration of 45 μg/mL.

A guar gum gel 1.5% (w/v) was prepared by dissolving 0.6 g of guar gum (Sigma-Aldrich, USA) in 40 mL of sterile distilled water and heat sterilized by autoclave. The solution of nisin was incorporated within the guar gum gel in a proportion of 1:1, obtaining a final 0.75% (w/v) biogel with 22.5 μg/mL of nisin.

Regarding antibiotics solutions, 6.6, 4.76 and 10.62 mg of Clindamycin (Cayman, USA), Gentamicin (PanReac AppliChem, USA) and Vancomycin (PanReac AppliChem, USA), respectively, were dissolved in 10 mL of sterile water and filtered through a 0.22 μm cellulose acetate membrane filter. Stock solutions were kept frozen at -80°C and diluted to the final concentrations of 0.033 μg/mL for clindamycin, 0.238 μg/mL for gentamicin and 0.531 μg/mL for vancomycin, prior to utilization.

### *In vitro* evaluation of the inhibitory action of combined antimicrobial

**Fig 1:**
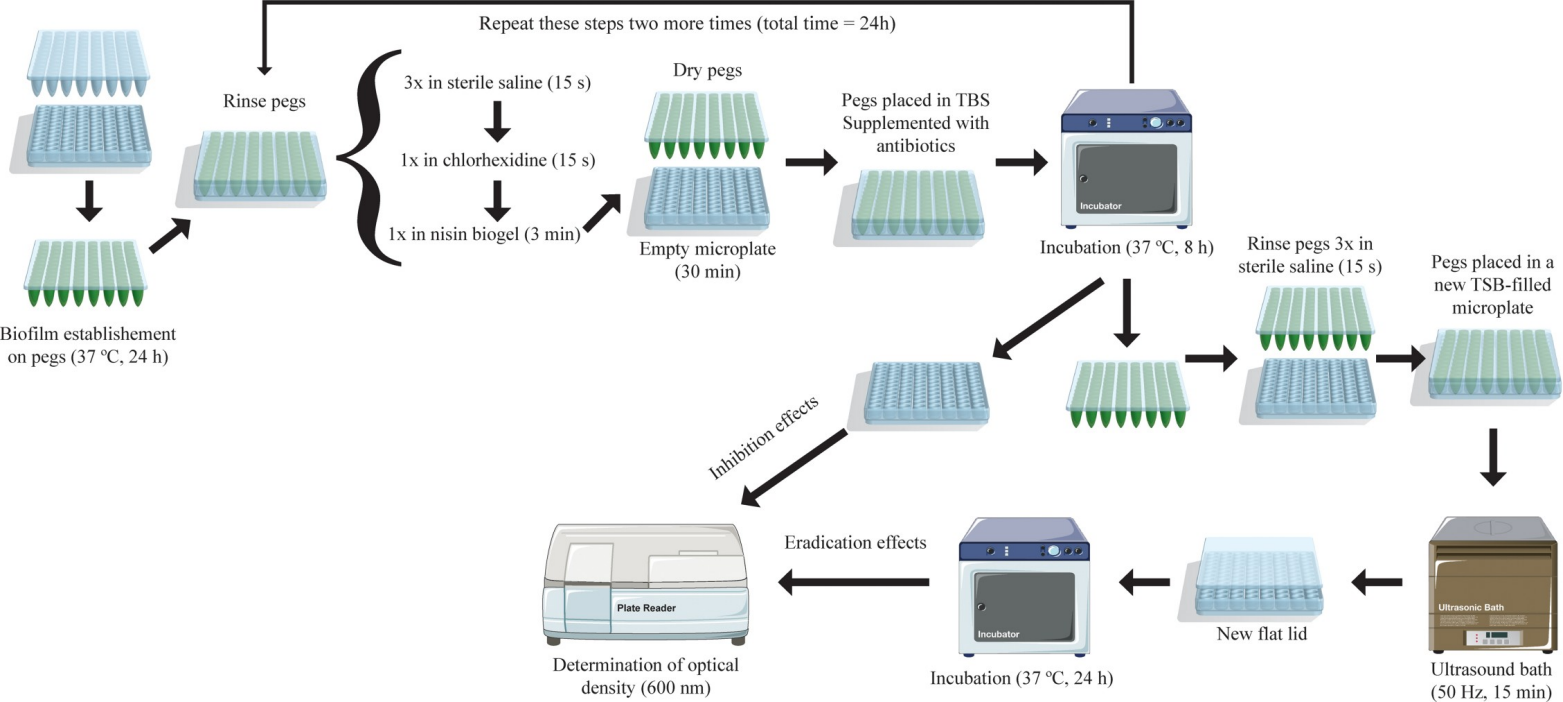
Schematic representation of the protocol developed to study the susceptibility of diabetic foot infection *Staphylococcus aureus* biofilms to different antimicrobial compounds combinations. The schematic representation shows the treatment combination when all three antimicrobials, chlorhexidine, nisin guar gum gel and antibiotics, are applied.

An innovative *in vitro* protocol (Fig 1) was designed to mimic currently accepted DFI therapeutic protocols, aiming at evaluating the combined action of the antiseptic chlorhexidine, the AMP nisin and the antibiotics clindamycin, gentamicin and vancomycin against the DFI staphylococci under study.

Strains were grown in a non-selective BHI agar medium at 37°C for 24 h. Bacterial suspensions of approximately 10^8^ c.f.u./mL were prepared directly from plate cultures using a 0.5 McFarland standard in sterile normal saline and then diluted in Tryptic Soy Broth (TSB) (VWR, Belgium) medium supplemented with 0.25% (w/v) glucose (Merck, USA), to a concentration of 10^6^ c.f.u./mL. A 200 μL volume of each bacterial suspension was distributed in a 96-well flat-bottomed polystyrene microtiter plate, covered with 96-peg polystyrene lid (Nunc, Thermo Fisher Scientific, Denmark) and incubated statically for 24 h at 37°C, to allow biofilm formation on the pegs surface. After establishment of *S*. *aureus* biofilms, the peg lid was rinsed periodically using different combinations of antiseptic, nisin, and antibiotics solutions, in order to evaluate the inhibitory potential of fifteen different combinations of antimicrobials, as follows: Chlorhexidine (Chx), nisin-biogel (NBG), nisin-biogel plus chlorhexidine (NBG+Chx), clindamycin (Cli), clindamycin plus chlorhexidine (Cli+Chx), clindamycin plus nisin-biogel (Cli+NBG), clindamycin plus chlorhexidine plus nisin-biogel (Cli+Chx+NBG), gentamicin (Gen), gentamicin plus chlorhexidine (Gen+Chx), gentamicin plus nisin-biogel (Gen+NBG), gentamicin plus chlorhexidine plus nisin-biogel (Gen+Chx+NBG), vancomycin (Van), vancomycin plus chlorhexidine (Van+Chx), vancomycin plus nisin-biogel (Van+NBG) and vancomycin plus chlorhexidine plus nisin-biogel (Van+Chx+NBG).

Positive (bacterial suspensions in broth medium with no antimicrobials) and negative (broth medium only) controls were also included in the assays.

The concentration of antimicrobials used corresponded to the MIC values obtained both in this experiment and in previous studies (Table 1).

**Table 1:** Minimum Inhibitory Concentration (MIC) Values of the Antimicrobial Solutions Chlorhexidine, Nisin-Biogel, Clindamycin, Gentamicin and Vancomycin Regarding the Diabetic Foot Infection *Staphylococcus aureus* Isolates Under Study.

Class	Antimicrobial	MIC (μg/mL)	Reference
Antiseptic	Chlorhexidine	6	Present study
Antimicrobial Peptide	Nisin-biogel	22.5	Santos et al., 2016
Antibiotic	Clindamycin	0.033	Mottola et al., 2016c
	Gentamicin	0.238	
	Vancomycin	0.531	

MIC, minimum inhibitory concentration

First, biofilm-covered peg lids were rinsed three times in 0.9% NaCl (w/v) for 15 seconds, to remove planktonic bacteria; then placed in chlorhexidine (6 μg/mL) during 15 seconds; then placed in the nisin-biogel (22.5 μg/mL) for 3 minutes; and finally incubated in an empty microplate during 30 minutes to allow the biogel to dry. Afterwards, peg lids were placed in 96-well flat-bottomed polystyrene microtiter plates containing fresh TSB + 0.25% glucose medium supplemented with the antibiotics clindamycin (0.033 μg/mL), gentamicin (0.238 μμg/mL) or vancomycin (0.531 μg/mL). Microplates were incubated at 37°C during 8 h, after which the protocol cycle was repeated. A total of three cycles were performed, corresponding to a 24 h period.

When a treatment combination did not include chlorhexidine or nisin-biogel, the peg lid was placed in an empty microplate during the corresponding incubation period. When a treatment combination did not include antibiotics, the peg lid was placed in non-supplemented TSB broth.

The inhibitory effect of the antimicrobials was determined by removing the peg lids and determining the optical density (OD) at 600 nm of the suspensions in the 96 well-plate using a microplate reader (BGM LABTECH, Germany). Then, the peg lids where rinsed three more times in 0.9% NaCl, placed in new microplates containing only 200 μL of fresh TSB + 0.25% glucose medium and incubated in an ultrasound bath (Grant MXB14, England), at 50 Hz for 15 minutes, in order to disperse the biofilm-based bacteria from the pegs surface. Afterwards, peg lids were discarded and microplates were covered with normal lids and incubated for 24 h at 37°C to allow the growth of surviving bacterial cells. The biofilm eradication effect was determined through measurement of the OD (600 nm) of these overnight suspensions.

Experiments were conducted in triplicate, and independent replicates were performed at least three times in different days.

### Statistical analysis

Statistical analysis was performed using the IBM SPSS Statistics V20 Software for Windows. Minimum, maximum, mean and standard deviation values were determined for all quantitative variables. Differences between MIC and MBC values were evaluated using the T-test.

Analysis of variance (ANOVA) for Randomized Complete Block Design (RCBD) was used to analyze the variables studied and post-hoc comparisons were assessed using Least Significant Differences tests. The OD results obtained in the biofilm inhibition and eradication assays were evaluated in order to determine the most effective combination of antimicrobial compounds. Each combination was considered a different treatment and all the *S*. *aureus* strains (each strain acting as a block) were exposed to all the different treatments. A two-tailed *p* value ≤ 0.05 was considered to be statistically significant in all the applied tests.

## Results

### Chlorhexidine MIC and MBC values

Chlorhexidine MIC and MBC values are presented in Table 2. MIC values ranged from 1.4 to 7.0 μg/mL, with an average value of 5.7±1.5 μg/mL; MBC values ranged from 9.8 to 68.8 μg/mL, with an average value of 15.5±14.9 μg/mL. MIC and MBC are statistically different (*p* value = 0.004), as determined through a paired sample T-test.

**Table 2:** Chlorhexidine Minimum Inhibitory Concentration (MIC) and Minimum Bactericidal Concentration (MBC) Values Regarding *Staphylococcus aureus* Diabetic Foot Infection Strains.

Strain (n = 24)		MIC (μg/mL)	MBC (μg/mL)
A 1.1	MRSA	5.6	9.8
A 5.2		4.2	9.8
A 6.3		4.2	39.2
B 3.2		5.6	9.8
B 3.3		5.6	9.8
B 7.3	MRSA; MDR	7.0	68.6
B 13.1	MRSA; MDR	7.0	9.8
B 14.2	MRSA; MDR	5.6	9.8
S 1.1	MRSA; MDR	7.0	19.6
S 2.2		7.0	9.8
S 3.1		7.0	9.8
S 5.2		4.2	9.8
S 12.2		1.4	9.8
S 14.1		4.2	9.8
S 16.1	MRSA; MDR	4.2	9.8
S 17.2		4.2	9.8
S 21.1	MRSA; MDR	7.0	9.8
S 21.3	MRSA; MDR	7.0	9.8
S 23.2		4.2	9.8
S 25.2		7.0	9.8
S 27.2		7.0	9.8
S 27.3		7.0	49.0
S 32.2		7.0	9.8
ATCC 29213		7.0	9.8
Mean		5.7	15.5
Minimum		1.4	9.8
Maximum		7.0	68.6
Std. Dev.		1.5	14.9

A, aspirate; ATCC, american type culture collection; B, biopsy; MDR, multidrug resistant; MRSA, methicillin resistant *Staphylococcus aureus*; S, swab; Std. Dev., standard deviation.

Antimicrobial agents are classified as bactericidal if the MBC value is no more than four times higher than their MIC value [35]. Chlorhexidine mean MBC was 2.72-fold higher than the mean MIC; therefore, chlorhexidine can be considered as a bactericidal agent against the *S*. *aureus* strains used in this study.

### *In vitro* evaluation of the inhibitory action of combined antimicrobials

**Fig 2:**
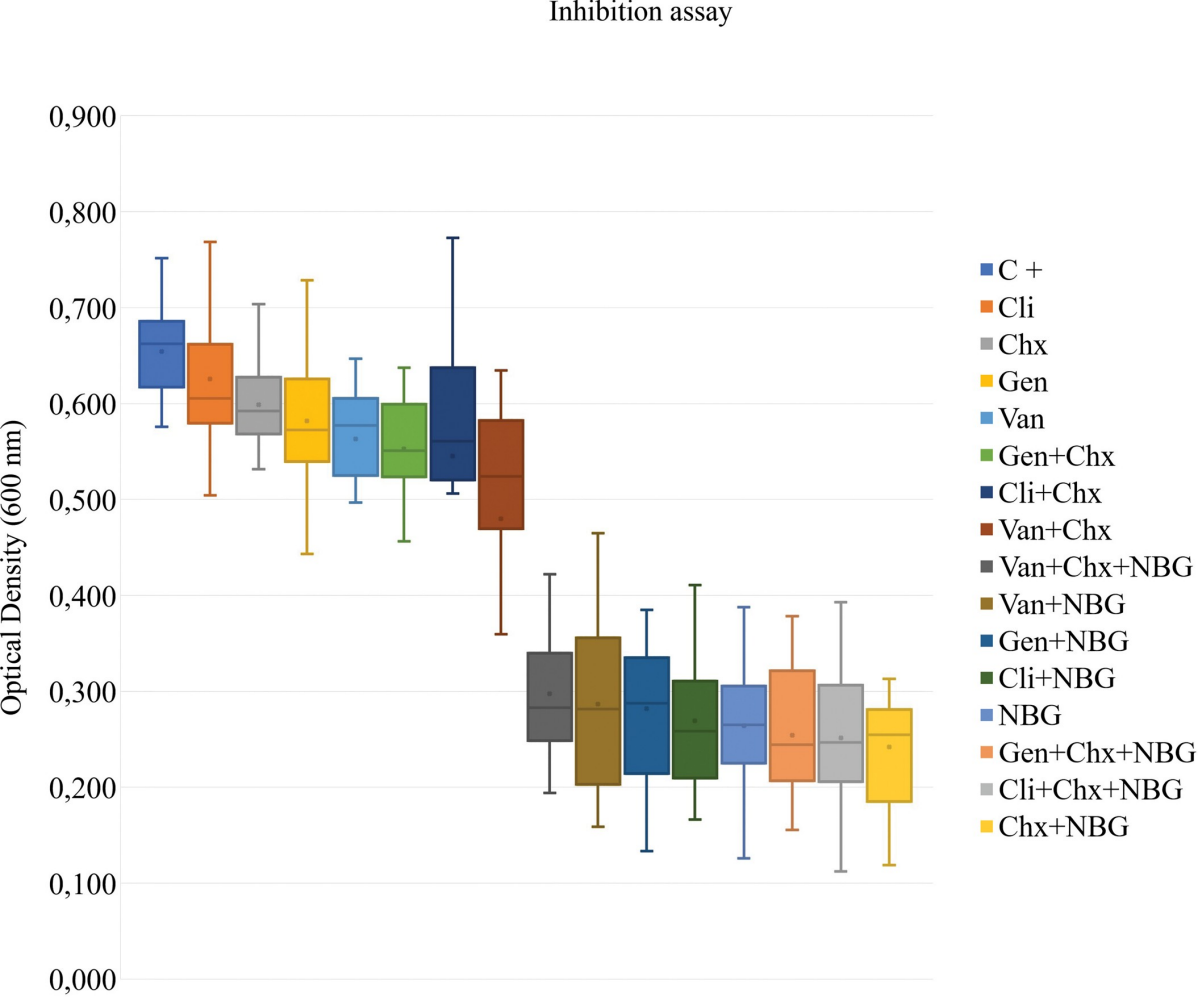
Inhibitory activity of antimicrobial compounds, alone or in combination, against biofilms formed by diabetic foot infection *Staphylococcus aureus* isolates. C +, positive control; Chx, chlorohexidine (6 μg/mL); Cli, clindamycin (0.033 μg/mL); Gen, gentamicin (0.238 μg/mL); NBG, nisin-biogel (22.5 μg/mL); Van, vancomycin (0.531 μg/mL). The means (x) and standard deviations of three independent determinations are presented. The negative control mean optical density value was 0.101.

**Fig 3:**
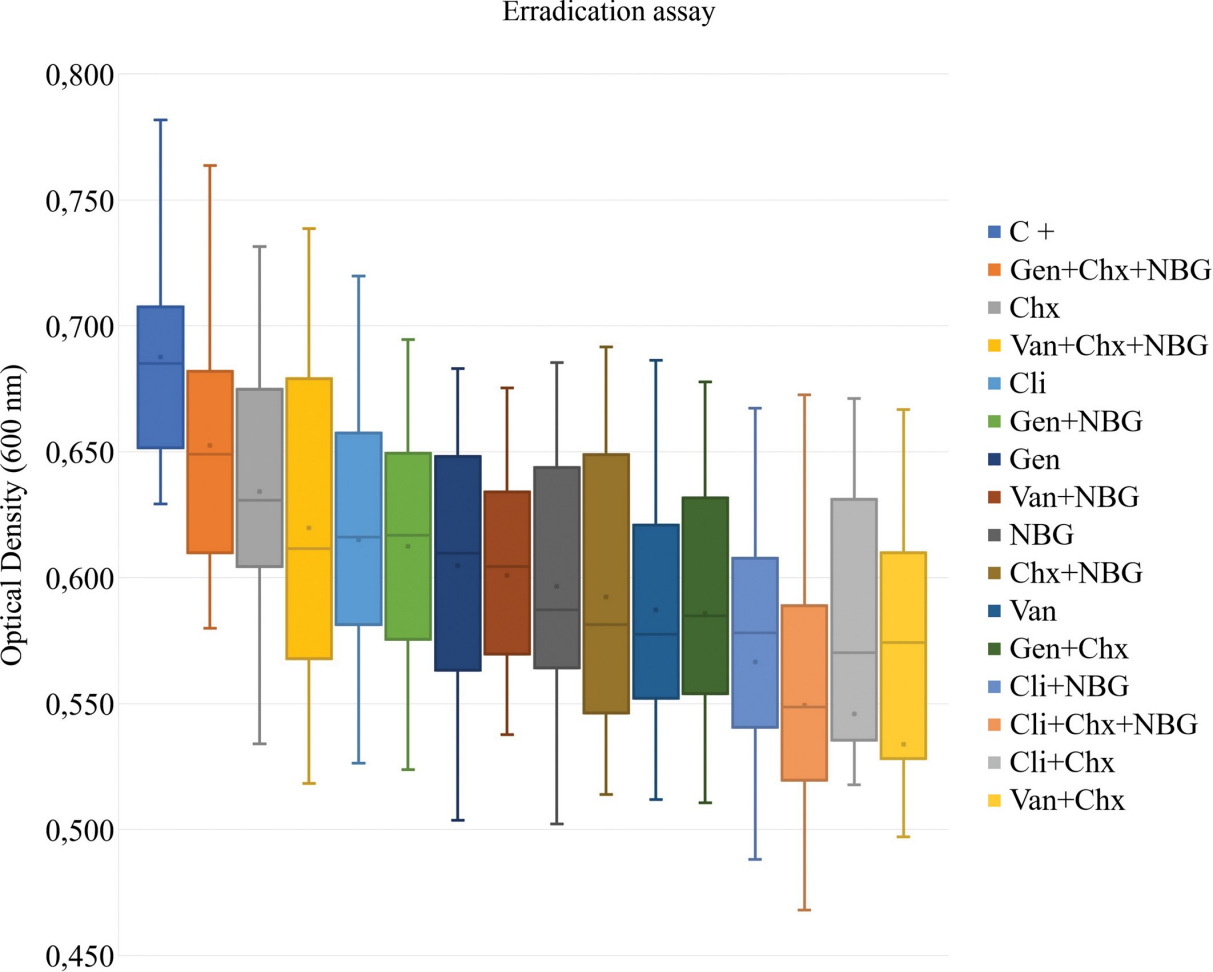
Eradication activity of different antimicrobial compounds, alone or in combination, against biofilms formed by diabetic foot infection *Staphylococcus aureus* isolates. C +, positive control; Chx, chlorohexidine (6 μg/mL); Cli, clindamycin (0.033 μg/mL); Gen, gentamicin (0.238 μg/mL); NBG, nisin-biogel (22.5 μg/mL); Van, vancomycin (0.531 μg/mL). The means (x) and standard deviations of three independent determinations are presented. The negative control mean optical density value was 0.101.

Growth rates were approximately the same between all strains under study. Considering that bacterial suspensions OD values are directly related to their biomass, the OD of each suspension after incubation with the different antimicrobial combinations was measured to compare their efficacy and to determine which antimicrobial combinations exhibited the higher biofilm inhibition and eradication levels (Figs 2 and 3; Tables 3 and 4).

**Table 3:** Inhibitory activity of different antimicrobial compounds combinations against diabetic foot infection *Staphylococcus aureus* biofilms.

A B	C+	Chx	NBG	Chx+ NBG	Cli	Cli+Chx	Cli+NBG	Cli+Chx+ NBG	Gen	Gen+Chx	Gen+ NBG	Gen+Chx+NBG	Van	Van+Chx	Van+ NBG	Van+Chx+NBG
**C+**	**A—B**	0.0551	0.3900	0.4122	0.0286	0.1086	0.3846	0.4027	0.0721	0.1014	0.3722	0.3997	0.0912	0.1744	0.3676	0.3568
	***p* value**	0.008	< 0.001	< 0.001	0.164	< 0.001	< 0.001	< 0.001	0.001	< 0.001	< 0.001	< 0.001	< 0.001	< 0.001	< 0.001	< 0.001
**Chx**			0.3349	0.3570	- 0.0264	0.0534	0.3294	0.3475	0.0169	0.0462	0.3170	0.3445	0.0360	0.1192	0.3124	0.3016
			< 0.001	< 0.001	0.199	0.010	< 0.001	< 0.001	0.409	0.025	< 0.001	< 0.001	0.081	< 0.001	< 0.001	< 0.001
**NBG**				0.0221	- 0.3614	- 0.2814	- 0.0054	0.0126	- 0.3179	- 0.2886	- 0.0178	0.0096	- 0.2988	- 0.2156	- 0.0224	- 0.0332
				0.283	< 0.001	< 0.001	0.791	0.539	< 0.001	< 0.001	0.385	0.640	< 0.001	< 0.001	0.276	0.107
**Chx+** **NBG**					- 0.3835	- 0.3035	- 0.0275	- 0.0094	- 0.3400	- 0.3107	- 0.0400	- 0.0124	- 0.3210	- 0.2378	- 0.0445	- 0.0553
					< 0.001	< 0.001	0.181	0.645	< 0.001	< 0.001	0.053	0.544	< 0.001	< 0.001	0.031	0.007
**Cli**						0.0799	0.3559	0.3740	0.0434	0.0727	0.3435	0.3710	0.0625	0.1457	0.3389	0.3281
						< 0.001	< 0.001	< 0.001	0.035	< 0.001	< 0.001	< 0.001	0.003	< 0.001	< 0.001	< 0.001
**Cli+Chx**							0.2759	0.2940	- 0.0364	- 0.0071	0.2635	0.2910	- 0.0174	0.0657	0.2590	0.2481
							< 0.001	< 0.001	0.077	0.727	< 0.001	< 0.001	0.397	0.002	< 0.001	< 0.001
**Cli+NBG**								0.0180	- 0.3124	- 0.2831	- 0.0124	0.0150	- 0.2934	- 0.2102	- 0.0169	- 0.0278
								0.380	< 0.001	< 0.001	0.546	0.464	< 0.001	< 0.001	0.410	0.177
**Cli+Chx+ NBG**									- 0.3305	- 0.3012	- 0.0305	- 0.0030	- 0.3115	- 0.2283	- 0.0350	- 0.0458
									< 0.001	< 0.001	0.139	0.884	< 0.001	< 0.001	0.089	0.026
**Gen**										0.0293	0.3000	0.3275	0.0190	0.1022	0.2955	0.2846
										0.155	< 0.001	< 0.001	0.355	< 0.001	< 0.001	< 0.001
**Gen+Chx**											0.2707	0.2982	- 0.0102	0.0729	0.2661	0.2553
											< 0.001	< 0.001	0.618	< 0.001	< 0.001	< 0.001
**Gen+** **NBG**												0.0275	- 0.2810	- 0.1977	- 0.0045	- 0.0153
												0.182	< 0.001	< 0.001	0.825	0.455
**Gen+Chx+NBG**													- 0.3085	- 0.2253	- 0.0320	- 0.0428
													< 0.001	< 0.001	0.120	0.038
**Van**														0.0832	0.2764	0.2656
														< 0.001	< 0.001	< 0.001
**Van+Chx**															0.1932	0.1824
															< 0.001	< 0.001
**Van+** **NBG**																- 0.0108
																0.599
**Van+Chx+NBG**																
															

Differences (A-B) between the optical density means presented by each treatment combination were assessed using Fisher’s least significant differences test. Significant differences (*p* ≤ 0.05) are highlighted (grey box). Chx, chlorohexidine (6 μg/mL); Cli, clindamycin (0.033 μg/mL); Gen, gentamicin (0.238 μg/mL); NBG, nisin-biogel (22.5 μg/mL); Van, vancomycin (0.531 μg/mL).

**Table 4:** Eradication activity of different antimicrobial compounds combinations against diabetic foot infection *Staphylococcus aureus* biofilms.

A B	C+	Chx	NBG	Chx+ NBG	Cli	Cli+Chx	Cli+NBG	Cli+Chx+ NBG	Gen	Gen+Chx	Gen+ NBG	Gen+Chx+NBG	Van	Van+Chx	Van+ NBG	Van+Chx+NBG
**C+**	**A—B**	0.0534	0.0910	0.0953	0.0726	0.1416	0.1210	0.1281	0.0827	0.1019	0.0751	0.0351	0.1004	0.1537	0.0867	0.0678
	***p* value**	0.002	< 0.001	< 0.001	< 0.001	< 0.001	< 0.001	< 0.001	< 0.001	< 0.001	< 0.001	0.036	< 0.001	< 0.001	< 0.001	< 0.001
**Chx**			0.0376	0.0419	0.0192	0.0882	0.0676	0.0847	0.0293	0.0485	0.0217	- 0.0182	0.0470	0.1003	0.0333	0.0144
			0.025	0.013	0.250	< 0.001	< 0.001	< 0.001	0.080	0.004	0.194	0.277	0.005	< 0.001	0.047	0.389
**NBG**				0.0043	- 0.0183	0.0506	0.0299	0.0471	- 0.0082	0.0109	- 0.0158	- 0.0558	0.0094	0.0627	- 0.0043	- 0.0231
				0.796	0.274	0.003	0.074	0.005	0.623	0.515	0.344	0.001	0.573	< 0.001	0.797	0.167
**Chx+** **NBG**					- 0.0226	0.0463	0.0256	0.0428	- 0.0125	0.0065	- 0.0201	- 0.0601	0.0051	0.0584	- 0.0086	- 0.0275
					0.176	0.006	0.126	0.011	0.453	0.694	0.229	0.001	0.760	0.001	0.606	0.101
**Cli**						0.0689	0.0483	0.0654	0.0100	0.0292	0.0024	- 0.0375	0.0277	0.0810	0.0140	- 0.0048
						< 0.001	0.004	< 0.001	0.547	0.081	0.882	0.026	0.098	< 0.001	0.402	0.772
**Cli+Chx**							- 0.0206	- 0.0034	- 0.0588	- 0.0397	- 0.0664	- 0.1064	- 0.0412	0.0120	- 0.0549	- 0.0738
							0.218	0.835	< 0.001	0.018	< 0.001	< 0.001	0.014	0.470	0.001	< 0.001
**Cli+NBG**								0.0171	- 0.0382	- 0.0190	- 0.0458	- 0.0858	- 0.0205	0.0327	- 0.0342	- 0.0531
								0.305	0.023	0.255	0.006	< 0.001	0.220	0.051	0.041	0.002
**Cli+Chx+ NBG**									- 0.0553	- 0.0362	- 0.0630	- 0.1029	- 0.0377	0.0155	- 0.0514	- 0.0703
									0.001	0.031	< 0.001	< 0.001	0.025	0.352	0.002	< 0.001
**Gen**										0.0191	- 0.0076	- 0.0476	0.0176	0.0709	0.0039	- 0.0149
										0.253	0.649	0.005	0.292	< 0.001	0.814	0.372
**Gen+Chx**											- 0.0267	- 0.0667	- 0.0014	0.0518	- 0.0152	- 0.0340
											0.111	< 0.001	0.929	0.002	0.364	0.042
**Gen+** **NBG**												- 0.0399	0.0252	0.0785	0.0115	- 0.0073
												0.017	0.132	< 0.001	0.490	0.662
**Gen+Chx+NBG**													0.0652	0.1185	0.0515	0.0326
													< 0.001	< 0.001	0.002	0.052
**Van**														0.0533	- 0.0137	- 0.0326
														0.002	0.412	0.052
**Van+Chx**															- 0.0670	- 0.0859
															< 0.001	< 0.001
**Van+** **NBG**																- 0.0188
																0.260
**Van+Chx+NBG**																
															

Differences (A-B) between the optical density means presented by each treatment combination were assessed using Fisher’s least significant differences test. Significant differences (*p* ≤ 0.05) are highlighted (grey box). Chx, chlorohexidine (6 μg/mL); Cli, clindamycin (0.033 μg/mL); Gen, gentamicin (0.238 μg/mL); NBG, nisin-biogel (22.5 μg/mL); Van, vancomycin (0.531 μg/mL).

First, inhibitory activity of the individual antimicrobial compounds alone was evaluated. Results showed that the nisin-biogel presented the highest level of biofilm inhibition, followed by the antibiotics vancomycin and gentamicin (Fig 2). Clindamycin had the lowest biofilm-inhibitory effect and no significant differences were detected between the OD of the suspension incubated with this antibiotic and the positive control (Table 3). When chlorhexidine was applied alone, its inhibitory activity against the biofilm-producing *S*. *aureus* strains was very similar to the inhibitory activity presented by the different antibiotics, as no significant differences were observed between results (*p* value > 0.05) (Table 3). Regarding the inhibitory action of the antimicrobial combinations tested, the higher inhibitory effect was presented by the combined application of chlorhexidine and nisin-biogel. Furthermore, when combined with the biogel, all antibiotics presented a significantly higher (*p* value < 0.05) antibiofilm ability (Fig 2, Table 3). No relevant differences were detected between the antibiotic resistant and the antibiotic susceptible strains under study. Treatment combinations that included nisin-biogel were the most effective regarding biofilm inhibition for all isolates tested (S1A Table).

Concerning the biofilm eradication assay, the OD values obtained after the application of the different antimicrobial compounds presented an uniform distribution and were significantly higher than those observed in the biofilm inhibition assay (Fig 3, Table 4). For individual compounds, the lowest OD values, which correspond to the highest eradication effect, were obtained after incubation with vancomycin, followed by incubation with nisin-biogel, gentamicin and clindamycin. There were no relevant differences between results, as all antimicrobial compounds presented a similar eradication effect of *S*. *aureus* biofilms. As observed in the biofilm inhibition results, no relevant differences were detected between antibiotic resistant and antibiotic susceptible strains under study (S1B Table).

Regarding biofilm eradication, results suggest that chlorhexidine and nisin-biogel increased the eradication potential of the other compounds, as the highest effects were presented by the following combinations: vancomycin plus chlorhexidine, clindamycin plus chlorhexidine, clindamycin plus chlorhexidine plus nisin-biogel and clindamycin plus nisin-biogel.

## Discussion

Diabetes *mellitus* is a serious public health problem, being one of four priority noncommunicable diseases [1]. Foot skin ulceration is one of the most frequent and costly complications of diabetes, being frequently infected by pathogenic microorganisms [6].

Diabetic foot infections have a multifactorial etiology, being *S*. *aureus* the most prevalent pathogen isolated from these wounds [4–5]. The emergence of antibiotic resistant and biofilm-forming *S*. *aureus* strains, together with the impairment of conventional antibiotic-based DFI therapeutics, emphasis the importance of developing novel therapeutic protocols for DFI management. This work analyzed the potential of the antiseptic chlorhexidine and the AMP nisin to be applied together with conventional antibiotics in DFI treatment.

Chlorhexidine is a widely used antiseptic agent with high antimicrobial activity [36]. Chlorhexidine MIC and MBC values obtained showed that chlorhexidine presented inhibitory and eradication action against the *S*. *aureus* strains under study at concentrations below 0.05% (500 μg/mL), the concentration established for wound cleansing [37–38].

The higher chlorhexidine MIC and MBC values regarding isolate B7.3 can be related to the fact of it being a MRSA and MDR strain. This strain harbors the antibiotic resistance gene *norA* [39], which presence is associated with increased resistance to antiseptic agents such as chlorhexidine [40]. Nonetheless, previous studies suggest that daily chlorhexidine bathing can reduce the acquisition of MRSA in intensive care unit patients [41]. In fact, chlorhexidine antimicrobial effects are persistent, mainly due to its ability to strongly bind to proteins present in the skin and mucosal surfaces [42]. The uptake of chlorhexidine by bacteria is extremely rapid, with a maximum effect occurring within 15 to 30 seconds [43] and, in contrast with other antiseptic agents, the residual antimicrobial activity of chlorhexidine is not affected by the presence of body fluids or blood [44]. Thus, chlorhexidine can be recommended for DFI wound cleansing.

The bacterial biofilm mode of growth is a major cause for the failure of conventional DFI antibiotherapy. It has been estimated that biofilm-based bacteria can tolerate antimicrobial agents at concentrations 10 to 1000-times higher than their genetically equivalent planktonic forms [45]. Since biofilms have a significant impact on public health, there is an urgent need for antibiofilm agents. Previous studies [46] suggest that nisin’s ability to form stable pores on prokaryotic membranes also occurs in biofilm-based bacteria, thus explaining its potent activity against *S*. *aureus* biofilms. Moreover, other studies reported an increase of the antimicrobial activity of antibiotics when combined with nisin [31]. Given that resistance to AMPs that target lipid II, such as nisin, does not develop easily [47], therapeutic protocols based on the combined administration of nisin with antibiotics may be an innovative strategy to control drug-resistant infections, such as DFIs.

This study evaluated the influence of chlorhexidine and the nisin-biogel in the inhibitory efficacy of conventional antibiotics against established biofilms formed by *S*. *aureus* DFI strains. As results demonstrate, individual antimicrobial compounds did not allow the complete elimination of the microorganisms, and the combination of different compounds resulted in an enhanced inhibitory efficacy against DFI pathogens.

Regarding biofilm inhibition, the combined action of the nisin-biogel and chlorhexidine showed the higher inhibitory effects. As observed for chlorhexidine, the nisin concentration required to inhibit biofilm cells was below its acceptable daily intake (0.13 mg/kg body weight) [48].

Results also showed that clindamycin and gentamicin biofilm inhibitory effects increased when combined with nisin. Both nisin and chlorhexidine exert their antimicrobial effect by disrupting the bacterial membrane [36,49], while clindamycin and gentamicin are antibiotics that inhibit protein synthesis. The application of nisin will allow the formation of stable pores in the bacterial membrane, allowing the antibiotic penetration to the bacterial cytoplasm, thus enabling them to act on bacterial ribosomes. Vancomycin biofilm inhibitory effects also increased when combined with this AMP. Although vancomycin and nisin are members of two different classes of antimicrobial agents, both target the essential cell wall precursor lipid II, blocking the cell wall biosynthesis [50]. These results are in agreement with previous studies that demonstrated synergistic relationships between conventional antibiotics and lantibiotics, such as nisin [31].

Bacteria embedded within biofilms are more persistent and difficult to eradicate [45], due to inefficient diffusion or sequestering of antibiotics within the biofilm matrix and also because biofilm-based bacterial cells tend to reduce their growth rate, protein synthesis and other physiologic activities, usually targeted by conventional antibiotic [51]. In fact, the low eradication effect observed for gentamicin can be related with the fact that aminoglycosides effectiveness relies heavily on bacterial growth phase and extra bacterial factors, such as oxygen availability, not maintained in the biofilm microenvironment [52].

A previous study conducted by our team demonstrated the capability of nisin to eradicate established *S*. *aureus* biofilms, even when incorporated in a guar gum gel [27,46]. The combination of different antimicrobial compounds allowed the higher eradication effects. Combinations of chlorhexidine plus antibiotics, nisin plus antibiotics, or even chlorhexidine plus nisin plus antibiotics, presented a higher eradication efficacy against DFI *S*. *aureus* strains than antibiotics alone. Also, since the nisin-biogel and chlorhexidine have a strong inhibitory and eradication effect against DFI *S*. *aureus* biofilms, these antimicrobial compounds could complement conventional antibiotherapy, enhancing antibiotics activity and possibly allowing to reduce the burden of antibiotic-resistant infections. Therefore, therapeutic protocols that include a first step of wound debridement, followed by antiseptic cleansing, AMP topical application and oral or systemic administration of antibiotics may represent the best approach to treat chronically infected skin ulcers and deserve further investigation aiming at their application to diabetic patients.

## Supporting information

S1 Table **Table A**. **Inhibitory activity of antimicrobial compounds, alone or in combination, against biofilms formed by diabetic foot infection *Staphylococcus aureus* isolates**.
Optical density values presented in the table were measured at 600 nm. The means and standard deviations of three independent determinations are presented. The negative control mean optical density value was 0.101.
C +, positive control; Chx, chlorohexidine (6 μg/mL); Cli, clindamycin (0.033 μg/mL); Gen, gentamicin (0.238 μg/mL); NBG, nisin-biogel (22.5 μg/mL); Van, vancomycin (0.531 μg/mL). A, aspirate; ATCC, american type culture collection; B, biopsy; S, swab; Std. Dev., standard deviation.
**Table B**. **Eradication activity of antimicrobial compounds, alone or in combination, against biofilms formed by diabetic foot infection *Staphylococcus aureus* isolates**.
Optical density values presented in the table were measured at 600 nm. The means and standard deviations of three independent determinations are presented. The negative control mean optical density value was 0.101.
C +, positive control; Chx, chlorohexidine (6 μg/mL); Cli, clindamycin (0.033 μg/mL); Gen, gentamicin (0.238 μg/mL); NBG, nisin-biogel (22.5 μg/mL); Van, vancomycin (0.531 μg/mL). A, aspirate; ATCC, american type culture collection; B, biopsy; S, swab; Std. Dev., standard deviation
(DOCX)

## References

[1] World Health Organization. Global report on diabetes. World Health Organization; 2016 ISBN: 9789241565257 10.2337/db15-0956

[2] Lipsky BA, Berendt AR, Cornia PB, Pile JC, Peters EJ, Armstrong DG, et al 2012 Infectious diseases society of america clinical practice guideline for the diagnosis and treatment of diabetic foot infections. Clin Infect Dis. 2012 6;54(12): e132–173. 10.1093/cid/cis346 22619242

[3] Armstrong DG, Cohen K, Courric S, Bharara M, Marston W. Diabetic foot ulcers and vascular insufficiency: our population has changed, but our methods have not. J Diabetes Sci Technol. 2011 11;5: 1591–1595. 10.1177/193229681100500636 22226282 PMC3262731

[4] Hobizal KB, Wukich DK. Diabetic foot infections: current concept review. Diabet Foot Ankle. 2012;3: 18409 10.3402/dfa.v3i0.18409 22577496 PMC3349147

[5] Mendes JJ, Marques-Costa A, Vilela C, Neves J, Candeias N, Cavaco-Silva P, et al Clinical and bacteriological survey of diabetic foot infections in Lisbon. Diabetes Res Clin Pract. 2012 1;95(1): 153–161. 10.1016/j.diabres.2011.10.001 22019426

[6] Lipsky BA, Aragón-Sánchez J, Diggle M, Embil J, Kono S, Lavery L, et al IWGDF guidance on the diagnosis and management of foot infections in persons with diabetes. Diabetes Metab Res Rev. 2016 1;32(S1): S45–74. 10.1002/dmrr.2699 26386266

[7] Dang C, Prasad Y, Boulton A, Jude E. Methicillin-resistant *Staphylococcus aureus* in the diabetic foot clinic: a worsening problem. Diabet Med. 2003 2;20: 159–161. 10.1046/j.1464-5491.2003.00860.x 12581269

[8] Kluytmans J, van Belkum A, Verbrugh H. Nasal carriage of *Staphylococcus aureus*: epidemiology, underlying mechanisms, and associated risks. Clin Microbiol Rev. 1997 7;10(3): 505–520. 9227864 10.1128/cmr.10.3.505 PMC172932

[9] Chambers HF, DeLeo FR. Waves of resistance: *Staphylococcus aureus* in the antibiotic era. Nat Rev Microbiol. 2009 9;7(9): 629–641. 10.1038/nrmicro2200 19680247 PMC2871281

[10] Mottola C, Semedo-Lemsaddek T, Mendes JJ, Melo-Cristino J, Tavares L, Cavaco-Silva P, et al Molecular typing, virulence traits and antimicrobial resistance of diabetic foot staphylococci. J Biomed Sci. 2016 3;8(23): 33 10.1186/s12929-016-0250-7 26952716 PMC4782296

[11] Akhi MT, Ghotaslou R, Memar MY, Asgharzadeh M, Varshochi M, Pirzadeh T, et al Frequency of MRSA in diabetic foot infections. Int J Diab Dev Ctries. 2017 3;37(1): 58–62. 10.1007/s13410-016-0492-7

[12] Dickschat JS. Quorum sensing and bacterial biofilms. Nat Prod Rep. 2010 3;27(3): 343–369. 10.1039/b804469b 20179876

[13] Vert M, Doi Y, Hellwich K, Hess M, Hodge P, Kubisa P, et al Terminology for biorelated polymers and applications (IUPAC Recomendations 2012). Pure Appl Chem. 2012;84(2): 377–410. 10.1351/PAC-REC-10-12-04

[14] Malik A, Mohammad Z, Ahmad J. The diabetic foot infections: bioflms and antimicrobial resistance. Diabetes Metab Syndr. 2013;7(2): 101–107. 10.1016/j.dsx.2013.02.006 23680250

[15] Lipsky BA, Hoey C, Cruciani M, Mengoli C. Topical antimicrobial agents for preventing and treating foot infections in people with diabetes (Protocol). Cochrane Database Syst Rev. 2014;3: CD011038 10.1002/14651858.CD011038 PMC6481886 28613416

[16] Schlett CD, Millar EV, Crawford KB, Cui T, Lanier JB, Tribble DR, et al Prevalence of chlorhexidine-resistant methicillin-resistant *Staphylococcus aureus* following prolonged exposure. Antimicrob Agents Chemoter. 2014 8;58(8): 4404–4410. 10.1128/AAC.02419-14 24841265 PMC4136006

[17] Bonez PC, Alves CF, Dalmolin TV, Agertt VA, Midzal CR, Flores C, et al Chlorhexidine activity against bacterial biofilms. Am J Infect Control. 2013 12;41(12): e119–122. 10.1016/j.ajic.2013.05.002 23910527

[18] Touzel RE, Sutton JM, Wand ME. Establishment of a multi-species biofilm model to evaluate chlorhexidine efficacy. J Hosp Infect. 2016 2;92(2): 154–160. 10.1016/j.jhin.2015.09.013 26597632

[19] Chidiac C, Bru J, Choutet P, Decazes J, Dubreuil L, Leport C, et al Management of diabetic foot infections. Med Mal Infect. 2007 1;37: 14–25. 10.1016/j.medmal.2006.10.001

[20] Bader M. Diabetic foot infection. Am Fam Physician. 2008 7;78(1): 71–79. 18649613

[21] Duarte N, Gonçalves A. Pé diabético. Angiol Cir Vasc. 2011;7(2): 65–79.

[22] Binda E, Marinelli F, Marcone GL. Old and new glycopeptide antibiotics: Action and resistance. Antibiotics (Basel). 2014 11;3(4): 572–594. 10.3390/antibiotics3040572 27025757 PMC4790382

[23] Lipsky BA, Hoey C. Topical antimicrobial therapy for treating chronic wounds. Clin Infect Dis. 2009 11;49: 1541–1549. 10.1086/644732 19842981

[24] Hancock RE, Sahl HG. Antimicrobial and host-defense peptides as new anti-infective therapeutic strategies. Nat Biotechnol. 2006 12;24(12): 1551–1557. 10.1038/nbt1267 17160061

[25] Rosenfeld Y, Papo N, Shai Y. Endotoxin (lipopolysaccharide) neutralization by innate immunity host-defense peptides. Peptide properties and plausible modes of action. J Biol Chem. 2006 1;281(3): 1636–1643. 10.1074/jbc.M504327200 16293630

[26] Batoni G, Maisetta G, Esin S. Antimicrobial peptides and their interaction with biofilms of medically relevant bacteria. Biochim Biophys Acta. 2016 5;1858(5): 1044–1060. 10.1016/j.bbamem.2015.10.013 26525663

[27] Santos R, Gomes D, Macedo H, Barros D, Tibério C, Veiga AS, et al Guar gum as a new antimicrobial peptide delivery system against diabetic foot ulcers *Staphylococcus aureus* isolates. J Med Microbiol. 2016 10;65(10): 1092–1099. 10.1099/jmm.0.000329 27498987

[28] Zhu M, Liu P, Niu Z. A perspective on general direction and challenges facing antimicrobial peptides. Chinese Chem Lett. 2017;28(4): 703–708. 10.1016/j.cclet.2016.10.001

[29] Santos S, Semedo-Lemsaddek T, Oliveira M. Bacteriocins In: Oliveira M, Serrano I, editors. The challenges of antibiotic resistance in the development of new therapeutics. Book Series: Frontiers in Antimicrobial Agents. Bentham Science Publishers Ltd; 2015 pp. 178–207. 10.2174/97816810814031150101

[30] O’Driscoll NH, Labovitiadi O, Cushnie TP, Matthews KH, Mercer DK, Lamb AJ. Production and evaluation of an antimicrobial peptide-containing wafer formulation for topical application. Curr Microbiol. 2013 3;66(3): 271–278. 10.1007/s00284-012-0268-3 23183933

[31] Mataraci E, Dosler S. *In vitro* activities of antibiotics and antimicrobial cationic peptides alone and in combination against methicillin-resistant *Staphylococcus aureus* biofilms. Antimicrob Agents Chemother. 2012 12; 56(12): 6366–6371. 10.1128/AAC.01180-12 23070152 PMC3497160

[32] Mottola C, Mendes JJ, Cristino JM, Cavaco-Silva P, Tavares L, Oliveira M. Polymicrobial biofilms by diabetic foot clinical isolates. Folia Microbiol (Praha). 2016 1;61(1): 35–43. 10.1007/s12223-015-0401-3 26104539

[33] Clinical and Laboratory Standards Institute. Methods for Dilution Antimicrobial Susceptibility Tests for Bacteria That Grow Aerobically; CLSI document M07-A10. 10th ed Wayne, PA: Clinical and Laboratory Standards Institute; 2015.

[34] Clinical and Laboratory Standards Institute. Methods for Determining Bactericidal Activity of Antimicrobial Agents; CLSI document M26-A. 1st ed Wayne, PA: Clinical and Laboratory Standards Institute; 1999.

[35] French GL. Bactericidal agents in the treatment of MRSA infections–the potential role of daptomycin. J Antimicrob Chemother. 2006 12;58(6): 1107–1117. 10.1093/jac/dkl393 17040922

[36] Milstone AM, Passaretti C, Perl TM. Chlorhexidine: Expanding the armamentarium for infection control and prevention. Clin Infect Dis. 2008 1;46(2): 274–281. 10.1086/524736 18171263

[37] Main RC. Should chlorhexidine gluconate be used in wound cleansing?. J Wound Care. 2008 3; 17(3): 112–114. 10.12968/jowc.2008.17.3.28668 18376652

[38] Wound Healing and Management Node Group. Evidence summary: Wound management–Chlorhexidine. Wound Pract Res. 2017;25(1): 49–51.

[39] Mottola C, Matias CS, Mendes JJ, Melo-Cristino J, Tavares L, Cavaco-Silva P, et al Susceptibility patterns of *Staphylococcus aureus* biofilms in diabetic foot infections. BMC Microbiol. 2016 6;16(1): 199 10.1186/s12866-016-0812-6 27339028 PMC4918071

[40] Liu Q, Zhao H, Han Z, Shu W, Wu Q, Ni Y. Frequency of biocide-resistant genes and susceptibility to chlorhexidine in high-level mupirocin-resistant *Staphylococcus aureus* (MuH MRSA). Diagn Microbiol Infect Dis. 2015 8;82(4): 278–283. 10.1016/j.diagmicrobio.2015.03.023 26008124

[41] Climo MW, Sepkowitz KA, Zuccotti G, Fraser VJ, Warren DK, Perl TM, et al The effect of daily bathing with chlorhexidine on the acquisition of methicillin-resistant *Staphylococcus aureus*, vancomycin-resistant *Enterococcus*, and healthcare-associated bloodstream infections: Results of a quasi-experimental multicenter trial. Crit Care Med. 2009 6;37(6): 1858–65. 10.1097/CCM.0b013e31819ffe6d 19384220

[42] Lim KS, Kam PC. Chlorhexidine–Pharmacology and clinical applications. Anaesth Intensive Care. 2008 7;36(4): 502–512. 10.1177/0310057X0803600404 18714617

[43] McDonnell G, Russel DA. Antiseptics and disinfectants: Activity, action, and resistance. Clin Microbiol Rev. 1999 1;12(1): 147–179. 9880479 10.1128/cmr.12.1.147 PMC88911

[44] Huang H, Chen B, Wang H, He M. The efficacy of daily chlorhexidine bathing for preventive healthcare-associated infections in adult intensive care units. Korean J Intern Med. 2016 11;31(6): 1159–1170. 10.3904/kjim.2015.240 27048258 PMC5094930

[45] Kaplan JB. Antibiotic-induced biofilm formation. Int J Artif Organs. 2011 9;34(9): 737–751. 10.5301/ijao.5000027 22094552

[46] Okuda K, Zendo T, Sugimoto S, Iwase T, Tajima A, Yamada S, et al Effects of bacteriocins on methicillin-resistant *Staphylococcus aureus* biofilm. Antimicrob Agents Chemother. 2013 11;57(11): 5572–5579. 10.1128/AAC.00888-13 23979748 PMC3811281

[47] Yeaman MR, Yount NY. Mechanisms of antimicrobial peptide action and resistance. Pharmacol Rev. 2003 3;55(1): 27–55. 10.1124/pr.55.1.2 12615953

[48] European Food Safety Authority, Younes M, Aggett P, Aguilar F, Crebelli R, Dusemund B, et al Safety of nisin (E 234) as a food additive in the light of new toxicological data and the proposed extension of use. EFSA Journal. 2017 12;15(12): 5063 10.2903/j.efsa.2017.5063 PMC7009836 32625365

[49] Wiedemann I, Breukink E, van Kraaij C, Kuipers OP, Bierbaum G, de Kruijff B, et al Specific binding of nisin to the peptidoglycan precursor lipid II combines pore formation and inhibition of cell wall biosynthesis for potent antibiotic activity. J. Biol. Chem. 2011 1;276(3): 1772–1779. 10.1074/jbc.M006770200 11038353

[50] Kohanski MA, Dwyer DJ, Collins JJ. How antibiotics kill bacteria: from targets to networks. Nat Rev Microbiol. 2010 6;8(6): 423–435. 10.1038/nrmicro2333 20440275 PMC2896384

[51] LaPlante KL, Mermel LA. *In vitro* activities of telavancin and vancomycin against biofilm-producing *Staphylococcus aureus*, *S*. *epidermidis*, and *Enterococcus faecalis* strains. Antimicrob Agents Chemother. 2009 7;53(7): 3166–3169. 10.1128/AAC.01642-08 19451302 PMC2704672

[52] Henry-Stanley MJ, Hess DJ, Wells CL. Aminoglycoside inhibition of *Staphylococcus aureus* biofilm formation is nutrient dependent. J Med Microbiol. 2014 6;63(6): 861–869. 10.1099/jmm.0.068130-0 24696518 PMC4030398

