# Supplementary material for: Diabetic foot infections: Application of a nisin-biogel to complement the activity of conventional antibiotics and antiseptics against Staphylococcus aureus biofilms
Source: PLoS One. 2019 Jul 24;14(7):e0220000. doi: 10.1371/journal.pone.0220000 (PMC6655664; doi:10.1371/journal.pone.0220000)
Supplement: S1 Table — Table A. Inhibitory activity of antimicrobial compounds, alone or in combination, against biofilms formed by diabetic foot infection Staphylococcus aureus isolates. Optical density values presented in the table were measured at 600 nm. The means and standard deviations of three independent determinations are presented. The negative control mean optical density value was 0.101. C +, positive control; Chx, chlorohexidine (6 μg/mL); Cli, clindamycin (0.033 μg/mL); Gen, gentamicin (0.238 μg/mL); NBG, nisin-biogel (22.5 μg/mL); Van, vancomycin (0.531 μg/mL). A, aspirate; ATCC, american type culture collection; B, biopsy; S, swab; Std. Dev., standard deviation. Table B. Eradication activity of antimicrobial compounds, alone or in combination, against biofilms formed by diabetic foot infection Staphylococcus aureus isolates. Optical density values presented in the table were measured at 600 nm. The means and standard deviations of three independent determinations are presented. The negative control mean optical density value was 0.101. C +, positive control; Chx, chlorohexidine (6 μg/mL); Cli, clindamycin (0.033 μg/mL); Gen, gentamicin (0.238 μg/mL); NBG, nisin-biogel (22.5 μg/mL); Van, vancomycin (0.531 μg/mL). A, aspirate; ATCC, american type culture collection; B, biopsy; S, swab; Std. Dev., standard deviation (DOCX) [file pone.0220000.s001.docx]

| **Strain (n=24)** | **C +** | **Chx** | **NBG** | **Chx + NBG** | **Cli** | **Cli + Chx** | **Cli + NBG** | **Cli + Chx + NBG** | **Gen** | **Gen + Chx** | **Gen + NBG** | **Gen + Chx + NBG** | **Van** | **Van + Chx** | **Van + NBG** | **Van + Chx + NBG** |
| --- | --- | --- | --- | --- | --- | --- | --- | --- | --- | --- | --- | --- | --- | --- | --- | --- |
| A 1.1 | 0.585 | 0.587 | 0.293 | 0.270 | 0.596 | 0.560 | 0.207 | 0.253 | 0.531 | 0.538 | 0.358 | 0.202 | 0.509 | 0.360 | 0.174 | 0.249 |
| A 5.2 | 0.624 | 0.572 | 0.292 | 0.252 | 0.545 | 0.506 | 0.216 | 0.219 | 0.571 | 0.572 | 0.291 | 0.221 | 0.498 | 0.447 | 0.179 | 0.235 |
| A 6.3 | 0.722 | 0.613 | 0.388 | 0.308 | 0.769 | 0.765 | 0.411 | 0.393 | 0.686 | 0.626 | 0.385 | 0.261 | 0.597 | 0.608 | 0.321 | 0.271 |
| B 3.2 | 0.658 | 0.595 | 0.312 | 0.276 | 0.601 | 0.562 | 0.284 | 0.206 | 0.539 | 0.570 | 0.278 | 0.211 | 0.583 | 0.504 | 0.356 | 0.280 |
| B 3.3 | 0.661 | 0.696 | 0.311 | 0.297 | 0.611 | 0.517 | 0.235 | 0.207 | 0.589 | 0.637 | 0.367 | 0.191 | 0.631 | 0.514 | 0.229 | 0.237 |
| B 7.3 | 0.576 | 0.571 | 0.228 | 0.222 | 0.548 | 0.526 | 0.193 | 0.201 | 0.503 | 0.550 | 0.254 | 0.212 | 0.497 | 0.529 | 0.196 | 0.286 |
| B 13.1 | 0.663 | 0.602 | 0.223 | 0.192 | 0.621 | 0.686 | 0.392 | 0.333 | 0.574 | 0.525 | 0.205 | 0.326 | 0.564 | 0.522 | 0.364 | 0.339 |
| B 14.2 | 0.666 | 0.594 | 0.201 | 0.183 | 0.668 | 0.620 | 0.248 | 0.268 | 0.632 | 0.589 | 0.210 | 0.264 | 0.540 | 0.591 | 0.276 | 0.265 |
| S 1.1 | 0.681 | 0.668 | 0.252 | 0.177 | 0.595 | 0.562 | 0.221 | 0.235 | 0.648 | 0.594 | 0.175 | 0.254 | 0.607 | 0.557 | 0.190 | 0.250 |
| S 2.2 | 0.594 | 0.588 | 0.211 | 0.183 | 0.591 | 0.518 | 0.204 | 0.208 | 0.521 | 0.501 | 0.222 | 0.206 | 0.501 | 0.508 | 0.254 | 0.194 |
| S 3.1 | 0.687 | 0.704 | 0.292 | 0.312 | 0.651 | 0.745 | 0.306 | 0.291 | 0.729 | 0.726 | 0.363 | 0.314 | 0.647 | 0.630 | 0.308 | 0.302 |
| S 5.2 | 0.660 | 0.568 | 0.231 | 0.223 | 0.580 | 0.543 | 0.384 | 0.327 | 0.570 | 0.605 | 0.332 | 0.379 | 0.576 | 0.623 | 0.382 | 0.383 |
| S 12.2 | 0.724 | 0.452 | 0.253 | 0.257 | 0.570 | 0.201 | 0.177 | 0.112 | 0.649 | 0.552 | 0.336 | 0.270 | 0.520 | 0.085 | 0.235 | 0.318 |
| S 14.1 | 0.667 | 0.542 | 0.224 | 0.168 | 0.617 | 0.143 | 0.268 | 0.208 | 0.533 | 0.523 | 0.211 | 0.234 | 0.601 | 0.081 | 0.287 | 0.324 |
| S 16.1 | 0.629 | 0.532 | 0.173 | 0.173 | 0.577 | 0.082 | 0.257 | 0.204 | 0.557 | 0.279 | 0.208 | 0.183 | 0.571 | 0.076 | 0.302 | 0.340 |
| S 17.2 | 0.660 | 0.548 | 0.257 | 0.251 | 0.622 | 0.553 | 0.281 | 0.300 | 0.566 | 0.528 | 0.315 | 0.223 | 0.581 | 0.537 | 0.339 | 0.247 |
| S 21.1 | 0.604 | 0.591 | 0.289 | 0.282 | 0.593 | 0.602 | 0.188 | 0.183 | 0.543 | 0.544 | 0.355 | 0.197 | 0.578 | 0.557 | 0.205 | 0.220 |
| S 21.3 | 0.675 | 0.610 | 0.268 | 0.263 | 0.666 | 0.595 | 0.260 | 0.260 | 0.542 | 0.516 | 0.308 | 0.262 | 0.559 | 0.520 | 0.269 | 0.266 |
| S 23.2 | 0.667 | 0.574 | 0.126 | 0.119 | 0.704 | 0.600 | 0.313 | 0.240 | 0.600 | 0.558 | 0.133 | 0.156 | 0.611 | 0.527 | 0.355 | 0.369 |
| S 25.2 | 0.495 | 0.560 | 0.262 | 0.238 | 0.505 | 0.557 | 0.238 | 0.267 | 0.443 | 0.457 | 0.249 | 0.210 | 0.367 | 0.458 | 0.203 | 0.327 |
| S 27.2 | 0.706 | 0.630 | 0.294 | 0.293 | 0.613 | 0.643 | 0.293 | 0.309 | 0.582 | 0.537 | 0.285 | 0.324 | 0.596 | 0.549 | 0.431 | 0.422 |
| S 27.3 | 0.752 | 0.681 | 0.316 | 0.313 | 0.767 | 0.773 | 0.396 | 0.325 | 0.657 | 0.615 | 0.325 | 0.341 | 0.611 | 0.594 | 0.465 | 0.389 |
| S 32.2 | 0.727 | 0.679 | 0.333 | 0.278 | 0.826 | 0.681 | 0.331 | 0.333 | 0.608 | 0.601 | 0.318 | 0.345 | 0.546 | 0.635 | 0.399 | 0.365 |
| ATCC 29213 | 0.615 | 0.621 | 0.309 | 0.277 | 0.581 | 0.555 | 0.167 | 0.154 | 0.597 | 0.523 | 0.283 | 0.325 | 0.621 | 0.505 | 0.159 | 0.262 |
| **Mean** | **0.654** | **0.599** | **0.264** | **0.242** | **0.625** | **0.545** | **0.269** | **0.251** | **0.582** | **0.553** | **0.282** | **0.254** | **0.563** | **0.480** | **0.286** | **0.297** |
| Std. Dev. | 0.057 | 0.058 | 0.057 | 0.054 | 0.076 | 0.175 | 0.072 | 0.066 | 0.063 | 0.080 | 0.068 | 0.061 | 0.060 | 0.166 | 0.087 | 0.060 |

**Table 1.** **Inhibitory activity of antimicrobial compounds, alone or in combination, against biofilms formed by diabetic foot infection *Staphylococcus aureus* isolates.** Optical density values presented in the table were measured at 600 nm. The means and standard deviations of three independent determinations are presented. The negative control mean optical density value was 0.101. C +, positive control; Chx, chlorohexidine (6 μg/mL); Cli, clindamycin (0.033 µg/mL); Gen, gentamicin (0.238 µg/mL); NBG, nisin-biogel (22.5 µg/mL); Van, vancomycin (0.531 µg/mL). A, aspirate; ATCC, american type culture collection; B, biopsy; S, swab; Std. Dev., standard deviation.

| **Strain (n=24)** | **C +** | **Chx** | **NBG** | **Chx + NBG** | **Cli** | **Cli + Chx** | **Cli + NBG** | **Cli + Chx + NBG** | **Gen** | **Gen + Chx** | **Gen + NBG** | **Gen + Chx + NBG** | **Van** | **Van + Chx** | **Van + NBG** | **Van + Chx + NBG** |
| --- | --- | --- | --- | --- | --- | --- | --- | --- | --- | --- | --- | --- | --- | --- | --- | --- |
| A 1.1 | 0.637 | 0.600 | 0.541 | 0.530 | 0.570 | 0.606 | 0.548 | 0.561 | 0.562 | 0.547 | 0.565 | 0.587 | 0.573 | 0.604 | 0.569 | 0.583 |
| A 5.2 | 0.629 | 0.612 | 0.564 | 0.582 | 0.581 | 0.570 | 0.602 | 0.539 | 0.565 | 0.568 | 0.620 | 0.638 | 0.585 | 0.572 | 0.558 | 0.546 |
| A 6.3 | 0.711 | 0.659 | 0.647 | 0.620 | 0.644 | 0.633 | 0.610 | 0.588 | 0.625 | 0.619 | 0.651 | 0.688 | 0.598 | 0.613 | 0.611 | 0.613 |
| B 3.2 | 0.648 | 0.534 | 0.502 | 0.514 | 0.613 | 0.571 | 0.588 | 0.569 | 0.504 | 0.527 | 0.554 | 0.613 | 0.567 | 0.587 | 0.607 | 0.636 |
| B 3.3 | 0.673 | 0.689 | 0.569 | 0.594 | 0.526 | 0.518 | 0.489 | 0.468 | 0.622 | 0.562 | 0.623 | 0.671 | 0.522 | 0.511 | 0.538 | 0.560 |
| B 7.3 | 0.680 | 0.623 | 0.588 | 0.584 | 0.578 | 0.557 | 0.540 | 0.524 | 0.569 | 0.579 | 0.612 | 0.632 | 0.552 | 0.566 | 0.634 | 0.682 |
| B 13.1 | 0.676 | 0.605 | 0.562 | 0.535 | 0.659 | 0.657 | 0.613 | 0.610 | 0.642 | 0.586 | 0.551 | 0.609 | 0.684 | 0.590 | 0.636 | 0.604 |
| B 14.2 | 0.686 | 0.637 | 0.614 | 0.653 | 0.633 | 0.667 | 0.574 | 0.555 | 0.659 | 0.585 | 0.646 | 0.585 | 0.553 | 0.588 | 0.617 | 0.594 |
| S 1.1 | 0.733 | 0.694 | 0.676 | 0.675 | 0.711 | 0.671 | 0.660 | 0.673 | 0.683 | 0.678 | 0.695 | 0.674 | 0.666 | 0.667 | 0.675 | 0.683 |
| S 2.2 | 0.639 | 0.556 | 0.538 | 0.544 | 0.585 | 0.555 | 0.568 | 0.617 | 0.558 | 0.540 | 0.570 | 0.607 | 0.550 | 0.576 | 0.578 | 0.523 |
| S 3.1 | 0.714 | 0.732 | 0.685 | 0.692 | 0.570 | 0.569 | 0.582 | 0.549 | 0.673 | 0.592 | 0.657 | 0.682 | 0.559 | 0.556 | 0.570 | 0.618 |
| S 5.2 | 0.688 | 0.673 | 0.669 | 0.650 | 0.666 | 0.644 | 0.617 | 0.589 | 0.632 | 0.639 | 0.643 | 0.671 | 0.608 | 0.612 | 0.606 | 0.655 |
| S 12.2 | 0.782 | 0.670 | 0.593 | 0.542 | 0.425 | 0.193 | 0.360 | 0.390 | 0.598 | 0.640 | 0.614 | 0.733 | 0.660 | 0.190 | 0.603 | 0.730 |
| S 14.1 | 0.662 | 0.625 | 0.582 | 0.553 | 0.641 | 0.236 | 0.510 | 0.537 | 0.564 | 0.585 | 0.606 | 0.669 | 0.547 | 0.209 | 0.570 | 0.611 |
| S 16.1 | 0.687 | 0.639 | 0.584 | 0.578 | 0.662 | 0.216 | 0.602 | 0.640 | 0.596 | 0.432 | 0.622 | 0.623 | 0.611 | 0.200 | 0.634 | 0.657 |
| S 17.2 | 0.636 | 0.538 | 0.522 | 0.528 | 0.582 | 0.550 | 0.543 | 0.519 | 0.511 | 0.511 | 0.524 | 0.583 | 0.514 | 0.540 | 0.570 | 0.570 |
| S 21.1 | 0.685 | 0.598 | 0.595 | 0.581 | 0.619 | 0.532 | 0.531 | 0.503 | 0.578 | 0.582 | 0.622 | 0.641 | 0.512 | 0.497 | 0.595 | 0.518 |
| S 21.3 | 0.641 | 0.611 | 0.566 | 0.556 | 0.591 | 0.542 | 0.550 | 0.476 | 0.560 | 0.551 | 0.540 | 0.580 | 0.555 | 0.527 | 0.568 | 0.572 |
| S 23.2 | 0.685 | 0.686 | 0.586 | 0.559 | 0.608 | 0.595 | 0.543 | 0.522 | 0.648 | 0.623 | 0.611 | 0.621 | 0.570 | 0.572 | 0.583 | 0.565 |
| S 25.2 | 0.692 | 0.604 | 0.581 | 0.651 | 0.599 | 0.533 | 0.488 | 0.576 | 0.648 | 0.625 | 0.592 | 0.657 | 0.582 | 0.533 | 0.556 | 0.567 |
| S 27.2 | 0.698 | 0.616 | 0.593 | 0.563 | 0.652 | 0.604 | 0.585 | 0.490 | 0.563 | 0.577 | 0.592 | 0.682 | 0.584 | 0.635 | 0.634 | 0.670 |
| S 27.3 | 0.806 | 0.692 | 0.646 | 0.647 | 0.720 | 0.658 | 0.630 | 0.600 | 0.679 | 0.639 | 0.678 | 0.756 | 0.631 | 0.632 | 0.642 | 0.695 |
| S 32.2 | 0.755 | 0.656 | 0.637 | 0.667 | 0.684 | 0.627 | 0.599 | 0.546 | 0.657 | 0.634 | 0.660 | 0.764 | 0.686 | 0.637 | 0.647 | 0.688 |
| ATCC 29213 | 0.661 | 0.676 | 0.678 | 0.619 | 0.639 | 0.601 | 0.667 | 0.548 | 0.623 | 0.639 | 0.653 | 0.693 | 0.624 | 0.601 | 0.622 | 0.739 |
| **Mean** | **0.688** | **0.634** | **0.597** | **0.592** | **0.615** | **0.546** | **0.567** | **0.549** | **0.605** | **0.586** | **0.612** | **0.652** | **0.587** | **0.534** | **0.601** | **0.620** |
| Std. Dev. | 0.045 | 0.051 | 0.051 | 0.053 | 0.062 | 0.136 | 0.065 | 0.061 | 0.051 | 0.053 | 0.045 | 0.052 | 0.050 | 0.136 | 0.035 | 0.063 |

**Table 2.** **Eradication activity of antimicrobial compounds, alone or in combination, against biofilms formed by diabetic foot infection *Staphylococcus aureus* isolates.** Optical density values presented in the table were measured at 600 nm. The means and standard deviations of three independent determinations are presented. The negative control mean optical density value was 0.101. C +, positive control; Chx, chlorohexidine (6 μg/mL); Cli, clindamycin (0.033 µg/mL); Gen, gentamicin (0.238 µg/mL); NBG, nisin-biogel (22.5 µg/mL); Van, vancomycin (0.531 µg/mL). A, aspirate; ATCC, american type culture collection; B, biopsy; S, swab; Std. Dev., standard deviation.
